# Epidemiology of traumatic brain injury in a South African major trauma cohort

**DOI:** 10.7189/jogh.16.04152

**Published:** 2026-05-15

**Authors:** Anne Ritter, Tahrir Rasool, Lani Finck, Julia M Dixon, Janette Verster, Hendrick J Lategan, Adriaan J Vlok, George Oosthuizen, Shaheem de Vries, Willem Stassen, Elmin Steyn, Craig Wylie, Dale A Barnhart, Nee-Kofi Mould-Millman, EpiC Study Site Collaborators, Mohammed Mayet, Lesley Hodsdon, L’Oreal Snyders, Leigh Wagner, Karlien Doubell, Denise Lourens

**Affiliations:** 1 Department of Emergency Medicine, Anschutz Medical Campus, University of Colorado, Aurora, Colorado, USA; 2 Collaborative for Emergency Care in Africa, Cape Town, South Africa; 3 Department of Pathology, Division of Forensic Medicine, Stellenbosch University, Cape Town, South Africa; 4 Department of Surgical Sciences, Division of Surgery, Stellenbosch University, Cape Town, South Africa; 5 Department of Surgical Sciences, Division of Neurosurgery, Stellenbosch University, Cape Town, South Africa; 6 Department of Family, Community, and Emergency Care, Division of Emergency Medicine, University of Cape Town, Cape Town, South Africa; 7 Emergency Medical Services, Western Cape Government Health and Wellness, Cape Town, South Africa

## Abstract

**Background:**

South Africa has one of the highest trauma burdens globally, with unique contextual challenges around health system capacity. Traumatic brain injury (TBI) is a significant contributor to post-injury mortality and morbidity. We aimed to examine TBI prevalence and outcomes in adult major trauma patients.

**Methods:**

We conducted a secondary analysis of the Epidemiology and Outcomes of Prolonged Trauma Care (EpiC) study, a prospective observational study of adult major trauma patients presenting to public-sector health facilities in South Africa’s Western Cape. Individuals injured from January 2022 to December 2024 with documentation of a head injury were included. We compared demographic, injury, and outcome variables across TBI categories: no TBI, mild (Glasgow Coma Scale (GCS) 13–15), moderate (GCS 9–12), severe (GCS 3–8). Logistic regression and Mantel-Haenszel analyses were used to examine mortality and neurofunctional disability.

**Results:**

We included 4815 patients within the EpiC database who had any head injury. Among those with TBI (n = 2297), 36.7% had mild, 39.6% moderate, and 23.7% severe TBI. A significantly higher percentage of severe TBI patients (n = 195; 35.8%) were transported directly to the tertiary facility. Overall, 7.2% of patients died before discharge from an EpiC site. Those with moderate (OR = 1.89; 95% confidence interval (CI) = 1.16–3.07) and severe (OR = 53.08; 95% CI = 37.61–74.91) TBI had higher odds of dying compared to those with no or mild TBI, controlling for age, multiple-trauma, and GCS qualifier. Among survivors, individuals with severe TBI (OR = 8.97; 95% CI = 5.45–14.74) had higher odds of poor neurofunctional recovery, measured by Glasgow Outcome at Discharge Scale, compared to those with no or mild TBI.

**Conclusions:**

TBI represents a significant burden in South Africa’s Western Cape. Within this head injury cohort, there are significant differences in injury characteristics and transport patterns. Mortality and neurofunctional recovery vary significantly across TBI severity but remain significantly worse for those with TBI even when controlling for potential confounders.

Globally, an estimated 4.4 million injury-related deaths occur annually, representing 8% of all deaths [1]. In addition to the high burden of mortality, injury-related morbidity negatively affects quality of life and imposes economic burden. Injury age-standardised disability adjusted life years (DALYs) are estimated at 3099 per 100 000, or 248 million DALYs worldwide [2]. The burden of injury-related mortality and morbidity is unequally distributed across regions and income groups [3].

Traumatic brain injury (TBI) contributes significantly to all-cause injury mortality and morbidity. Globally, TBI incidence is 939 cases per 100 000 people, or 69 million TBIs each year [4]. Most TBI cases are mild (81%), though the burden of moderate (11%) and severe (8%) TBI is significant; 5.5 million people experience moderate or severe TBI annually [4]. Mechanism of injury and TBI severity vary significantly between country income levels [5].

South Africa bears one of the highest trauma burdens globally; injuries account for an estimated 2.3 million DALYs. Interpersonal violence (6.5%) and road traffic injuries (3.0%) are among the top single leading causes of DALYs [6] and the most common causes of TBI [5]. TBI represents a significant burden on morbidity, healthcare utilisation, and economic strain [6,7]. Despite our understanding that TBI is a major challenge, the contribution of TBI to the overall burden of injury and outcomes among trauma patients varies widely. Currently, little is known about the contributions and epidemiology of TBI in South Africa.

The public trauma care system in South Africa is structured across district, regional, and limited tertiary centres. District hospitals receive referrals from surrounding primary/community health care centres, are mandated to provide emergency care, and may offer limited specialist services. Regional hospitals provide broader specialist services, including emergency care and radiological imaging. Tertiary hospitals deliver advanced specialist and subspecialist care, including intensive care, and accept referrals from district and regional hospitals [8].

Prehospital services, including transport from scene and interfacility transport, are delivered by provincially managed emergency medical services (EMS) agencies, which are frequently under-resourced. While EMS protocols prioritise transport of severely injured patients to the highest level of care available, systemic delays and geographic barriers remain significant obstacles to timely and effective trauma care [9].

Therefore, we aimed to determine the prevalence and outcomes of TBI in an adult major trauma cohort in the Western Cape of South Africa.

## METHODS

### Population/environment

The Epidemiology and Outcomes of Prolonged Trauma Care (EpiC) study began in 2021 to address knowledge gaps in the epidemiology and outcomes of trauma in the setting of potential delays to definitive care. EpiC is a multi-institutional, prospective, longitudinal study incorporating prehospital, hospital, and mortality data in the Western Cape region of South Africa. There are 12 study sites, all public-sector facilities, including one tertiary trauma centre (in Tygerberg), one rural district hospital (in Ceres), one urban district hospital (in Khayelitsha), one rural regional hospital (in Worcester), two community health centres (in Delft and Khayelitsha Site B), four government ambulance bases, and two Forensic Pathology Service laboratories [10]. Tygerberg Hospital provides speciality surgical and trauma care, receiving over 107 000 admissions per year [11], of which 12–15% are seriously injured [12].

We examined patients enrolled in the EpiC study from 1 January 2022 through 31 December 2024. The EpiC enrols individuals aged ≥18 years with a clinical encounter for traumatic injury at participating study sites [10]. EpiC excludes prisoners, patients who expire on-scene or during initial EMS transport, are transported via private EMS, whose injury occurred >24 hours before presentation at the first study site, or whose mechanism of injury was bite, sting, other envenomation, toxicologic injury, drowning, or hanging.

We examined EpiC cohort patients with any evidence of blunt and/or penetrating head injury defined as an Abbreviated Injury Score (AIS) head severity score of 1–6, wound care provided to the head, neurosurgical procedure, diagnosis of TBI or intracranial haemorrhage, abnormal head computed tomography (CT) (if available), and/or TBI-related mechanism of death. We excluded individuals for whom head injury status could not be classified.

### Data collection procedures

EpiC systematically collected data for all eligible trauma patients via chart abstraction from prehospital, in-hospital, and forensic pathology data. Research personnel captured patient data from initial presentation to a participating study site (including EMS) through all transfers to participating study sites until the end of care in a REDCap database [10]. We did not include data after discharge or transfer outside participating sites. Data from a complete postmortem report were available for review, as required for all unnatural deaths in South Africa [13]. Data for each patient was from a singular event resulting in traumatic injury.

### Variables and outcomes

We identified TBIs using AIS head score ≥2, worst 24-hour Glasgow Coma Scale (GCS), and head CT. We classified mild TBI as GCS 13–15, moderate as GCS 9–12 or moderate head CT finding regardless of AIS, and severe as GCS 3–8 or severe head CT finding regardless of AIS (Table S1 in the **Online Supplementary Document**). We defined individuals meeting inclusion criteria for the head injury cohort, but not meeting the above definitions of mild, moderate, or severe, as ‘no TBI’.

We calculated injury and clinical variables in accordance with the EpiC study data dictionary. We calculated hospital length of stay (LOS) as days until death in decedents and days until discharge (or lost) for survivors. We based substance use on documented substance abuse or alcohol use disorder present in the clinical chart. We determined GCS qualifier (yes/no) based on evidence of intoxication or presence of eye obstruction, chemical paralysis or sedation, or intubation. We examined injury severity variables (*e.g.* AIS, New Injury Severity Score (NISS), Shock Index (SI), Triage Early Warning Scale, South African Triage Scale (SATS)). If multiple data points were available for an injury severity variable, we documented the worst measurement within the first 24 hours of injury (*e.g.* highest SI, worst SATS).

To assess neurofunctional disability among survivors, we abstracted the Glasgow Outcome at Discharge Scale (GODS) in a subset of patients admitted to Tygerberg Hospital with a confirmed or suspected head injury who survived to discharge. We assigned GODS scores a numeric value increasing from 1 (‘Dead’) to 8 (‘Upper Good Recovery’).

All descendants received autopsies, which we used to determine the physiologic mechanism of death. TBI-related mechanisms of death included catastrophic destruction of the central nervous system tissue, including the brainstem.

### Statistical methods

We calculated the prevalence of TBI overall and by TBI severity. We compared demographics, injury characteristics, and outcomes by severity (no, mild, moderate, or severe TBI) using Kruskal-Wallis, χ^2^, or Fisher Exact tests. We compared EMS transport and time to facilities by survival status stratified by TBI severity.

The primary outcome of interest was all-cause mortality. We estimated the association between TBI severity and mortality using odds ratios (ORs) and Mantel-Haenszel adjusted ORs (aORs) adjusted for age (<40 *vs.* ≥40 years), multiple-trauma (SI≥1.4 or AIS non-head severity ≥3 *vs.* neither), and GCS qualifier. We similarly estimated the crude and adjusted association between TBI severity and neurofunctional disability dichotomised into ‘good’ recovery or ‘poor’ (moderate or severe disability, unconscious) among a subset of patients with valid GODS. We assessed the comparability of this subset to the larger head injury population by comparing demographic and injury characteristics among those not admitted to Tygerberg, those admitted to Tygerberg with GODS, and those admitted to Tygerberg without GODS.

We used SAS, version 9.4 (Cary, North Carolina, USA) for all analyses.

## RESULTS

**Figure 1:**
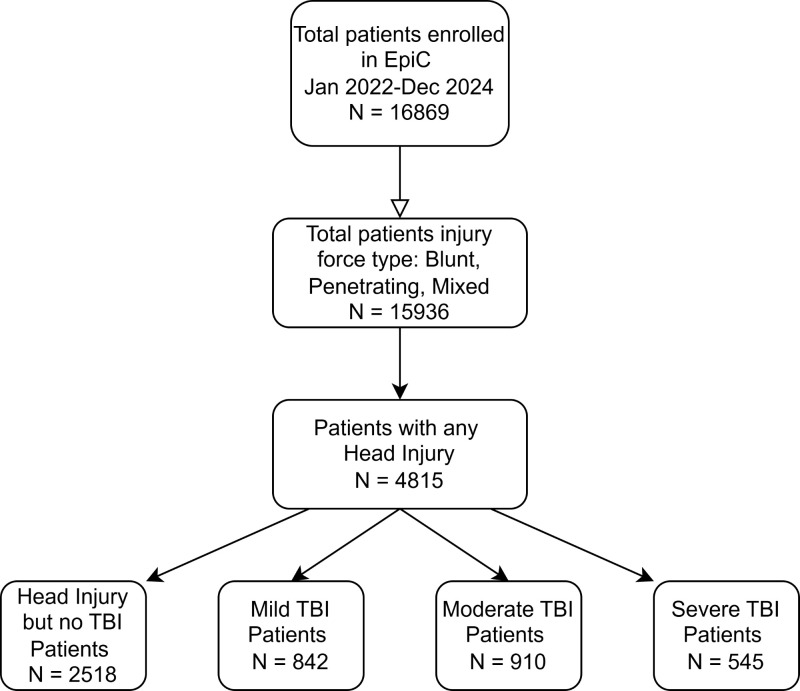
Enrolment diagram. EpiC – Epidemiology and Outcomes of Prolonged Trauma Care, TBI – traumatic brain injury.

Of the 16 869 patients enrolled in the EpiC study, we included 4815 (28.5%) who had documentation of any head injury (**Figure 1**). Among head injury patients, there were 2297 (47.7%) patients with any TBI, of whom 36.7% had mild, 39.6% moderate, and 23.7% severe TBI (Table S2 in the **Online Supplementary Document**).

Patients were predominantly young to middle-aged (median (MD) = 32.0; interquartile range (IQR) = 26.1–39.6) males (83.8%) with blunt injury (62.3%) from being struck/hit (47.8%). Compared to other TBI categories, more individuals with severe TBI had unintentional injury (38.9%), often from vehicular injuries (37.1%) (**Table 1**).

**Table 1:** Demographic characteristics of study participants*

	All (n = 4815)	No TBI (n = 2518)	Mild TBI (n = 842)	Moderate TBI (n = 910)	Severe TBI (n = 545)	*P*-value†
**Patient’s sex**						<0.0001
Male	4033 (83.8)	2040 (80.4)	1297 (86.4)	273 (91.6)	423 (88.5)	
Female	782 (16.2)	498 (19.8)	135 (16.0)	87 (9.6)	62 (11.4)	
**Age, MD (IQR)**	32.0 (26.1–39.6)	31.9 (26.1–39.8)	32.1 (26.1–39.5)	32.2 (26.1–40.1)	32.4 (25.8–38.6)	0.8788
**Substance abuse or alcohol use disorder**	499 (10.4)	291 (11.6)	98 (11.6)	77 (8.5)	33 (6.1)	0.0002
**GCS qualifier for the worst GCS in the first 24 h**	33 (1.5)	9 (0.9)	4 (1.1)	6 (1.5)	14 (4.6)	<0.0001
**Intoxication confirmed or suspected at the time of injury**	1524 (31.7)	821 (32.6)	269 (31.9)	308 (33.8)	126 (23.1)	<0.0001
**Mechanism of injury**						<0.0001
Firearm	222 (4.6)	61 (2.4)	33 (3.9)	43 (4.7)	85 (15.6)	
Struck/hit	2303 (47.8)	1163 (46.2)	441 (52.4)	497 (54.6)	202 (37.1)	
Stabbing or cut	1191 (24.7)	862 (34.2)	173 (20.5)	126 (13.8)	30 (5.5)	
Vehicular Injury	887 (18.4)	337 (13.4)	154 (18.3)	193 (21.2)	203 (37.2)	
Fall	166 (3.4)	68 (2.7)	36 (4.3)	43 (4.7)	19 (3.5)	
Other	46 (1.0)	27 (1.1)	5 (0.6)	8 (0.9)	6 (1.1)	
**Injury force type**						<0.0001
Blunt	3001 (62.3)	1376 (54.6)	551 (65.4)	663 (72.9)	411 (75.4)	
Penetrating	1220 (25.3)	825 (32.8)	160 (19.0)	128 (14.1)	107 (19.6)	
Blunt and penetrating	594 (12.3)	317 (12.6)	131 (15.6)	119 (13.1)	27 (5.0)	
**Intent of injury**						<0.0001
Unintentional or accidental	1075 (22.3)	420 (16.7)	212 (25.2)	231 (25.4)	212 (38.9)	
Intentional (self-harm or suicide)	17 (0.4)	12 (0.5)	1 (0.1)	2 (0.2)	2 (0.4)	
Intentional (assault/homicide)	3671 (76.2)	2067 (82.1)	625 (74.2)	665 (73.1)	314 (57.6)	
Other/undetermined	52 (1.1)	19 (0.7)	4 (0.5)	12 (1.3)	17 (3.1)	
**Primary EMS transport**	2217 (46.0)	1076 (42.7)	379 (45.0)	406 (44.6)	356 (65.3)	<0.0001
**Total hours from injury to first healthcare encounter, MD (IQR)**	2.0 (0.5–2.9)	2.0 (0.7–3.6)	2.0 (0.6–3.3)	1.8 (0.5–2.3)	0.6 (0.5–2.0)	<0.0001
n (missing)	4814 (1)	2518 (0)	841 (1)	910 (0)	545 (0)	
**Total hours from injury to first facility arrival, MD (IQR)**	2.0 (1.3–3.6)	2.0 (1.4–4.1)	2.0 (1.4–4.0)	2.0 (1.2–3.2)	1.6 (1.0–2.2)	<0.0001
n (missing)	4814 (1)	2518 (0)	841 (1)	910 (0)	545 (0)	
**First facility tier**						<0.0001
Primary	872 (18.1)	380 (15.1)	174 (20.7)	242 (26.6)	76 (13.9)	
Secondary district	841 (17.5)	595 (23.6)	138 (16.4)	64 (7.0)	44 (8.1)	
Secondary regional	2473 (51.4)	1384 (55.0)	450 (53.4)	409 (44.9)	230 (42.2)	
Tertiary	629 (13.1)	159 (6.3)	80 (9.5)	195 (21.4)	195 (35.8)	
**Total hours from injury to highest facility arrival, MD (IQR)‡**	4.8 (2.0–12.0)	3.5 (2.0–9.4)	6.2 (2.0–13.7)	9.4 (3.0–16.0)	3.9 (1.6–10.0)	<0.0001
N (Missing)	4814 (1)	2518 (0)	841 (1)	910 (0)	545 (0)	
**Highest facility tier**						<0.0001
Primary	12 (0.2)	5 (0.2)	0 (0.0)	0 (0.0)	7 (1.3)	
Secondary district	621 (12.9)	525 (20.8)	83 (9.9)	0 (0.0)	13 (2.4)	
Secondary regional	1862 (38.7)	1289 (51.2)	340 (40.4)	145 (15.9)	88 (16.1)	
Tertiary	2320 (48.2)	699 (27.8)	419 (49.8)	765 (84.1)	437 (80.2)	
**Experienced >1 facility transfer**	57 (1.2)	16 (0.6)	8 (1.0)	25 (2.8)	8 (1.5)	<0.0001
**Experienced ≥1 facility transfer**	1890 (39.3)	671 (26.6)	372 (44.2)	595 (65.4)	252 (46.2)	<0.0001
**NISS, MD (IQR)**	9.0 (3.0–22.0)	3.0 (2.0–6.0)	9.0 (6.0–17.0)	22.0 (17.0–27.0)	34.0 (25.0–50.0)	<0.0001
**SI≥1.4 within 24 h of injury**	115 (3.5)	42 (2.8)	7 (1.2)	21 (2.8)	45 (9.5)	<0.0001
n (missing)	3312 (1503)	1488 (1030)	605 (237)	745 (165)	474 (71)	
**Highest TEWS Score, MD (IQR)**	4.0 (3.0–6.0)	4.0 (3.0–5.0)	4.0 (2.0–5.0)	5.0 (3.0–6.0)	8.0 (6.0–10.0)	<0.0001
**Worst SATS**						<0.0001
Red	1032 (21.4)	300 (11.9)	103 (12.2)	199 (21.9)	430 (78.9)	
Orange	2470 (51.3)	1389 (55.2)	482 (57.2)	505 (55.5)	94 (17.2)	
Yellow	1219 (25.3)	783 (31.1)	232 (27.6)	185 (20.3)	19 (3.5)	
Green	94 (2.0)	46 (1.8)	25 (3.0)	21 (2.3)	2 (0.4)	
**Highest AIS head severity score, MD (IQR)**	1.0 (1.0–3.0)	1.0 (1.0–1.0)	2.0 (2.0–3.0)	3.0 (3.0–3.0)	4.0 (3.0–5.0)	<0.0001
n (missing)	4665 (150)	2370 (148)	842 (0)	908 (2)	545 (0)	
**AIS non-head severity ≥3**	474 (9.8)	215 (8.5)	66 (7.8)	82 (9.0)	111 (20.4)	<0.0001
**Head CT performed**	2267 (47.1)	500 (19.9)	469 (55.7)	892 (98.0)	406 (74.5)	<0.0001

AIS – Abbreviated Injury Scale, CT – computed tomography, EMS – emergency medical services, IQR – interquartile range, MD – median, NISS – New Injury Severity Score, SATS – South African Triage Scale, SI – Shock Index, TBI – traumatic brain injury, TEWS – Triage Early Warning Scale

*All values are presented as numbers (percentages) unless specified otherwise.

†*P*-values are from a χ^2^ test for categorical variables, and from the Kruskal-Wallis test for continuous variables.

‡Total hours from injury, inclusive of all transport time and time spent at prior facility(ies), as appropriate.

Overall, the cohort suffered minor anatomic injuries as defined by the NISS (MD = 9; IQR = 3–22), despite 72.7% having a red or orange worst SATS score within 24 hours post-injury (requiring care immediately or within ten minutes). However, NISS differed significantly by TBI severity. Those with severe TBI had NISS scores indicative of very severe injury (MD = 34; IQR = 25–50) and significantly greater prevalence of at least one non-head AIS body region score ≥3.

The percentage of individuals transported to their initial facility by EMS differed significantly across TBI categories and between survivors and decedents within the TBI category (Table S3 in the **Online Supplementary Document**). Approximately half of patients initially presented to the secondary regional hospital (51.4%), but 48.2% of the cohort received care at a tertiary hospital at some point (**Table 1**). Significantly more individuals with severe TBI were transported directly to a tertiary hospital (35.8%). Overall, 72.9% of those reaching the tertiary hospital were transferred from primary or secondary facilities.

More patients with severe TBI expired (49.7%) compared to other categories (<3%) (**Table 2**). The percentage of individuals with a physiologic mechanism of death attributed to brain injury increased significantly across worsening TBI categories. Haemorrhage or multiple organ failure/sepsis accounted for 20.5% of deaths among those with any TBI and was common among decedents with mild (20.0%), moderate (46.2%), and severe (18.1%) TBI.

**Table 2:** Outcomes among patients grouped by TBI severity*

	All (n = 4815)	No TBI (n = 2518)	Mild TBI (n = 842)	Moderate TBI (n = 910)	Severe TBI (n = 545)	*P*-value†
**Final disposition**						<0.0001
Deceased	346 (7.2)	39 (1.5)	10 (1.2)	26 (2.9)	271 (49.7)	
Discharged home (independent)	545 (11.3)	284 (11.3)	136 (16.2)	104 (11.4)	21 (3.9)	
Discharged home (f/u at lower level of care)	2477 (51.4)	1610 (63.9)	435 (51.7)	344 (37.8)	88 (16.1)	
Discharged home (f/u at speciality care)	921 (19.1)	415 (16.5)	184 (21.9)	255 (28.0)	67 (12.3)	
Discharge to hospice/palliative care	14 (0.3)	3 (0.1)	0 (0.0)	6 (0.7)	5 (0.9)	
Other	512 (10.6)	167 (6.6)	77 (9.1)	175 (19.2)	93 (17.1)	
**Mechanism of death**						<0.0001
Catastrophic tissue destruction	50 (14.5)	0 (0.0)	0 (0.0)	0 (0.0)	50 (18.5)	
CNS Injury	164 (47.4)	0 (0.0)	3 (30.0)	12 (46.2)	149 (55.2)	
Haemorrhage or exsanguination	38 (11.0)	17 (43.6)	0 (0.0)	2 (7.7)	19 (7.0)	
Multiple organ failure and sepsis	52 (15.1)	10 (25.6)	2 (20.0)	10 (38.5)	30 (11.1)	
Other	41 (11.9)	12 (30.8)	5 (50.0)	2 (7.7)	22 (8.1)	
**Any TBI-related mechanism of death**	221 (4.6)	0 (0.0)	4 (0.5)	13 (1.4)	204 (37.4)	<0.0001
**All: total hospital LOS in days, MD (IQR)**	1.4 (0.5–4.6)	0.8 (0.4–2.4)	1.4 (0.7–3.2)	4.1 (1.9–9.9)	4.4 (0.8–16.6)	<0.0001
**In survivors: total hospital LOS in days, MD (IQR)**	1.5 (0.5–4.6)	0.8 (0.4–2.4)	1.4 (0.7–3.2)	4.0 (1.9–9.7)	12.4 (4.6–25.4)	<0.0001
						
**In decedents: total hospital LOS in days/d until death, MD (IQR)**	0.9 (0.2–4.5)	0.3 (0.1–2.7)	2.0 (1.2–5.4)	6.6 (2.4–13.9)	0.8 (0.2–3.9)	<0.0001
**Death (all-cause) within 24 h**	175 (3.6)	25 (1.0)	2 (0.2)	0 (0.0)	148 (27.2)	<0.0001
**GCS at discharge in survivors, MD (IQR)**	15.0 (15.0–15.0)	15.0 (15.0–15.0)	15.0 (15.0–15.0)	15.0 (15.0–15.0)	15.0 (14.0–15.0)	<0.0001
n (missing)	4446 (369)	2470 (48)	831 (11)	876 (34)	269 (276)	
**GODS, MD (IQR)**	7.0 (5.0–8.0)	7.0 (6.0–8.0)	8.0 (7.0–8.0)	7.0 (6.0–8.0)	3.0 (1.0–6.0)	<0.0001
n (missing)	1090 (3725)	42 (2476)	211 (631)	540 (370)	297 (248)	

CNS – central nervous system, GCS – Glasgow Coma Scale, GODS – Glasgow Outcome at Discharge Scale, IQR – interquartile range, LOS – length of stay, MD – median, TBI – traumatic brain injury

*All values are presented as numbers (percentages) unless specified otherwise.

†*P*-values are from a χ^2^ test for categorical variables, and from the Kruskal-Wallis test for continuous variables.

Among survivors, the MD GCS at discharge was 15 for all categories. Final disposition differed significantly across TBI categories; more survivors with moderate (29.5%) and severe (26.3%) TBI required follow-up with speciality care or were discharged to hospice/palliative care compared to individuals with mild TBI (22.1%) and no TBI (16.8%).

Among survivors, the severe TBI group demonstrated the longest LOS (12.4 days). Among decedents, the MD LOS (*i.e.* days until death) was <1 for individuals with no TBI or severe TBI (**Table 2**). Overall, LOS was highly variable with significant outliers (Figure S1 in the **Online Supplementary Document**).

Individuals with mild TBI did not have statistically different odds of all-cause mortality compared to those without TBI (**Table 3**). However, the odds of mortality were twice as high among individuals with moderate TBI (OR = 1.9; 95% confidence interval (CI) = 1.2–3.1) compared to individuals with no or mild TBI in both unadjusted and adjusted analyses. The odds of mortality were over 50-fold higher among those with severe TBI (OR = 53.1; 95% CI = 37.6–74.9) compared to those with no or mild TBI in both unadjusted and adjusted analyses.

**Table 3:** ORs and aORs for all-cause mortality and neurofunctional disability

	All-cause mortality (n = 4815)			Neurofunctional disability (n = 941)*		
	**OR (95% CI)**	**OR (95% CI)**	**aOR (95% CI)†**	**OR (95% CI)**	**OR (95% CI)**	**aOR (95% CI)†**
**No TBI**	ref	ref	ref	ref	ref	ref
**Mild TBI**	0.76 (0.38–1.54)	ref	ref	0.29 (0.13–0.66)	ref	
**Moderate TBI**	1.87 (1.13–3.09)	1.99 (1.23–3.22)	1.89 (1.16–3.07)‡	0.76 (0.37–1.58)	2.01 (1.32–3.07)	2.06 (1.33–3.17)§
**Severe TBI**	62.87 (43.95–89.94)	66.83 (48.13–92.80)	53.08 (37.61–74.91)	3.48 (1.62–7.45)	9.18 (5.67–14.85)	8.97 (5.45–14.74)

aOR – adjusted odds ratio, CI – confidence interval, ref – reference, TBI – traumatic brain injury

*Neurofunctional disability was based on Glasgow Outcome at Discharge Scale (GODS), dichotomised into good (GODS = 7–8) and poor (GODS = 2–6); it was assessed among a subgroup of patients who survived to discharge and received a neurosurgical consult, neurosurgery, or were admitted by neurosurgery at Tygerberg Hospital.

†Mantel-Haenszel odds ratios adjusted for age (<40 *vs.* ≥40), evidence of multiple-trauma (SI ≥ 1.4 or AIS non-head severity ≥3 *vs.* no multiple-trauma), and evidence of intoxication or other GCS qualifier (yes *vs.* no).

‡No deaths were observed among moderate TBI patients with multiple-trauma and evidence of intoxication or a GCS qualifier; due to positivity violations in these strata, n = 125 excluded from the analysis.

§No disability observed among no/mild TBI patients <40 years with multiple-trauma and intoxication or among no/mild TBI patients ≥40 years with multiple-trauma and no intoxication; no good recovery observed among severe TBI patients with multiple-trauma and intoxication ≥40 years; n = 58 excluded for positivity.

**Figure 2:**
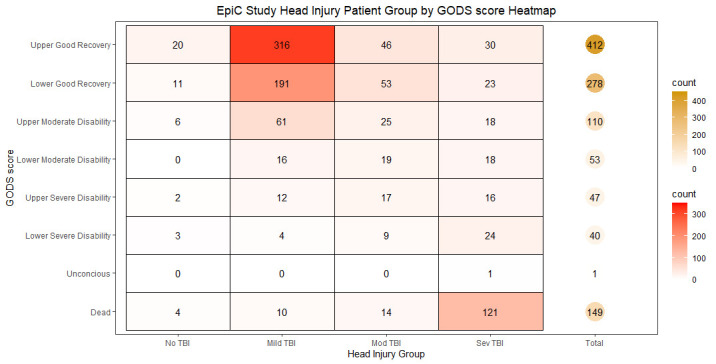
TBI related neurofunctional disability at discharge by head injury category for subset with GODS. EpiC – Epidemiology and Outcomes of Prolonged Trauma Care, GODS – Glasgow Outcome at Discharge Scale, TBI – traumatic brain injury.

Of those with GODS scores, severe TBI patients had the worst GODS score (MD = 3; IQR = 1–6). Approximately half of these severe TBI cases expired (48.2%), but 21.1% had ‘upper’ or ‘lower good recovery’ (GODS 7–8) upon discharge (**Figure 2**). When dichotomised into good (GODS 7–8) *vs.* poor (GODS 2–6) recovery, patients with severe TBI had significantly greater odds of poor recovery (OR = 3.48; 95% CI = 1.62–7.45) compared to individuals with no TBI (**Table 3**). After adjusting for age, multiple-trauma, and GCS qualifier, the odds of poor recovery were significantly greater for those with moderate (aOR = 2.06) and severe (aOR = 8.97) TBI compared to those with no or mild TBI (**Table 3**). There were notable differences between patients with and without GODS scores (Table S4 in the **Online Supplementary Document**).

## DISCUSSION

Reported TBI prevalence within trauma cohorts varies significantly worldwide (23–51%) [14–16]. In our current analysis of head injury patients in the EpiC study, the prevalence of any TBI (47.3%) is within previously reported ranges. In low- and middle-income countries, the distribution of TBI severity among TBI patients varies (23–82% mild, 9–35% moderate, 9–45% severe) [7,16–18]. Our data demonstrate a slightly higher prevalence of moderate TBI (39.6%) but are generally aligned with published ranges. Variability in TBI prevalence and distribution of TBI severity within trauma cohorts is multifactorial and stems from differences in the cohort methodology (*e.g.* enrolment criteria, variability in TBI definitions) and contextual factors (*e.g.* differential access to imaging, mechanisms of injury).

Severity classification of TBI in South Africa and in this study generally follows international clinical tools and standards [19]. However, at most district and regional hospitals, neuroimaging availability is limited, especially after hours, weekends, and public holidays, and is absent at some facilities [18,20]. Lack of or delayed imaging and reliance on GCS for clinical diagnosis may lead to delayed, missed, or underdiagnosis. Our analytic definitions of TBI included CT imaging when available and incorporating CT data increases estimates of TBI severity compared to the use of GCS alone. Only four deceased patients in our cohort with mild TBI (and none with no documented TBI) had a brain- or brainstem-related mechanism of death. We believe this is due to a lack of imaging (*e.g.* early death prior to imaging availability) rather than incomplete documentation. Underdiagnosis may also be more pronounced in patients with multi-system trauma or intoxication that obscures or mimics neurological deficits. After adjusting for the presence of intoxication or GCS qualifier in our cohort, individuals with moderate and severe TBI still demonstrated greater odds of dying compared to those with no or mild TBI, though the effect was attenuated in the severe TBI group (OR = 66.8; aOR = 53.1).

Contextual differences in the mechanism of injury and intent also contribute to variability in TBI prevalence and distribution of severity. Intentional injury contributes significantly to the overall burden of TBI in South Africa and is reported to be the highest contributor to injury DALYs (~ 1 million) [9]. Intentional injury accounts for ~ 70% of all TBIs in our analysis, drastically greater than in other countries (5.3–28%) [16,21,22]. Interpersonal violence in South Africa is significantly impacted by South Africa’s socioeconomic inequality, high unemployment, limited access to public services, and downstream consequences [23–26].

Delays in progression through the continuum of care are also rooted in contextual factors and may be associated with worse TBI outcomes. Socioeconomic factors, public health literacy, and limited ambulance availability may contribute to delays in laypersons seeking care [27]. Although Western Cape Direct Trauma Transfer guidelines recommend direct transport to neurosurgical facilities for severe head injuries, many patients, especially in rural or peri-urban areas, are first brought to the closest lower-level facilities or arrive as ‘walk-ins’ via police, family, or private transport. In our cohort, 65% of patients with severe TBI were transported by EMS, suggesting potential gaps in access to appropriate emergency services. In South Africa, timely progression through the continuum of care for trauma patients heavily relies on EMS ambulance transfer of patients to institutions with access to neuroimaging and neurosurgical capabilities [18]. However, delays with ambulance inter-facility transfer are exacerbated by structural inequities in the healthcare system and a large volume of trauma and TBI patients relative to public-sector health and EMS resources (*e.g.* limited EMS capacity and bed availability) [6,7]. Though a nuanced analysis of transport type and time from injury to facility is beyond the scope of the current report, our data demonstrate that primary EMS transport type and time from injury to the highest-level facility are associated with differences in mortality within TBI categories. Primary transport by EMS was associated with mortality, which may be attributed to greater injury severity in those initially transported by EMS. Time to the highest facility level was longer among survivors across all TBI categories. This is likely due to survivor bias (*e.g.* individuals who survive long enough to be transferred to a higher level of care take longer to reach their final facility).

In South Africa, neurosurgical services are concentrated in a limited number of tertiary institutions [6,16]. Specifically, CT imaging and neurosurgical expertise are largely absent at district and regional facilities [28]. This limits the ability to provide definitive TBI care where most patients in our cohort initially arrived; >40% of severe and 60% of moderate TBI required transfer for definitive neurotrauma care. Even when transferred, surgical intervention may be triaged based on resource constraints; theatre space and intensive care unit availability are prioritised for operable or salvageable cases, leaving some patients without surgical care [29,30]. Previous research from Cape Town indicates patients with moderate TBI have an average of 11 hours from injury to CT scan, and severe nine hours, with times tripling if they require neurosurgery [18]. Therefore, neurological observation becomes pertinent to assess for worsening of TBI symptoms. Systemic limitations in definitive neurotrauma care likely contribute to high reported rates of poor neurofunctional outcomes among TBI survivors [6,7,31], consistent with our head injury cohort.

While most patients were discharged home independently or with follow-up at non-speciality care, approximately half of the patients with severe TBI expired. This is on the high end of the range reported in previous studies of severe TBI in low- and middle-income countries (20–52%) [21,22,32,33]. Multiple factors may contribute to higher mortality rates, particularly the high percentage of individuals with severe TBI having a concomitant non-head AIS severity of ≥3 and shock index of ≥1.4, representing a significantly injured multiple-trauma group. The paucity of neurosurgeons, operating theatres, and neuro-intensive care relative to the large population of severe TBI patients likely contributes to high mortality [34].

In our analysis, MD LOS among survivors increased with TBI severity, and for severe TBI it was comparable to reports from other low- and middle-income countries (Uganda: seven days [35]; Nepal: >14 days [36]; Colombia: 17 days [37]). When only decedents are considered, the MD days until death for individuals with no TBI or severe TBI is >1 day. This is consistent with the leading mechanisms of death in each group – haemorrhage in patients without TBI (41%) and CNS injury in those with severe TBI (55%) – both of which are associated with rapid, non-preventable death. The LOS patterns vary and are shaped by resource limitations, trauma burden, and early in-hospital deaths. Variability in LOS in our study may reflect injury severity and systemic factors in South Africa’s public health system. In the Western Cape public healthcare sector, extrinsic (*e.g.* limited stepdown/rehab capacity, transport constraints) and intrinsic discharge barriers (*e.g.* inadequate discharge policies, postponed social worker placements) may prolong LOS [38]. Consequently, patients with residual neurological or functional impairments may experience extended hospitalisation [7,18]. Paradoxically, patients with less severe injuries may be observed without admission or more quickly discharged even if transferred to the tertiary hospital with neurosurgical service [18,31].

While the MD discharge GCS among survivors was 15 across all categories in our analysis, the cohort with GODS data reveals a more nuanced picture of neurofunctional disability. Individuals with severe (aOR = 9.0) or moderate (aOR = 2.1) TBI had significantly greater odds of poor recovery when compared to those with no or mild TBI after controlling for age, multiple-trauma, and GCS qualifier. Interestingly, we observed approximately 24% of survivors with no TBI had poor recovery (dichotomised GODS), potentially indicative of neurofunctional disability due to secondary neurologic insults from non-TBI injury mechanisms (*e.g.* sustained hypoxia and haemorrhagic hypotension). The GODS was only available for a non-representative sample and for a very small percentage (1%) of those with no TBI and must be interpreted with caution.

This study has several limitations. The EpiC study restricts inclusion to ‘major’ trauma. Therefore, TBI prevalence and outcomes cannot be generalised to the total population or a less severely injured cohort. Participating EpiC sites only include public facilities and EMS agencies, which limits generalisability to the private sector, particularly in South Africa. Third, EpiC excludes patients who expire prior to contact with the health system. This excludes the most severely injured, possibly those who expire due to catastrophic brain injury, creating survival bias. Additionally, EpiC excludes patients with injuries occurring more than 24 hours before presentation to a participating study site. This may exclude patients who otherwise meet ‘major’ trauma criteria but have survivable injuries, which limits population-level generalisability. Lastly, adjusted analyses of mortality and neurofunctional disability were limited by positivity violations, necessitating the dichotomisation of the GODS score and preventing adjustment for all confounders of interest.

## CONCLUSIONS

Head injury and TBI are significant contributors to major trauma and trauma-related morbidity and mortality in the Western Cape. Among all individuals with head injuries in the EpiC cohort, 47.7% had TBI, of whom 63% had moderate or severe TBI. Individuals with severe TBI had 53 times greater odds of all-cause mortality compared to those with no or mild TBI, even after adjusting for age, multiple-trauma, and GCS qualifier. Among survivors, there is variation in neurofunctional disability, but those with severe TBI demonstrate significantly worse GODS scores and have nine times greater odds of poor recovery compared to those with no or mild TBI after adjusting for age, multiple-trauma, and GCS qualifier. TBI epidemiology is impacted by delays to care and resource availability. Additional research on the type and timing of intervention in this head injury subpopulation of the EpiC cohort is required to further elucidate differences in mortality and neurofunctional outcome.

## Additional material


Online Supplementary Document
