# Supplementary material for: Epidemiology of traumatic brain injury in a South African major trauma cohort
Source: J Glob Health. 2026 May 15;16:04152. doi: 10.7189/jogh.16.04152 (PMC13178057; doi:10.7189/jogh.16.04152)
Supplement: Online Supplementary Document [file jogh-16-04152-s001.pdf]

Supplement to: Ritter A, Rasool T, Finck L, Dixon JD, Verster J, Lategan HJ, Vlok AJ, Oosthuizen, G, de Vries S, Stassen W, Steyn E, Wylie C; EpiC Study Site Collaborators, Barnhart DA, Mould-Millman NK. Epidemiology of traumatic brain injury in a South African major trauma cohort. J Glob Health. 2026;16:04152.

Supplemental Table 1: TBI Severity Criteria

TBI Severity Criteria: Head AIS severity score ≥2				
	**Mild TBI**	**Moderate TBI**	**Severe TBI**	**Variable Rules**
Glasgow Coma Score (GCS)	13 – 15	9 – 12	≤8	Lowest GCS in first 24 hours
Injury on computed tomography of brain		Intracranial hemorrhage, skull fracture, parenchymal injury or edema	Diffuse axonal injury, >5mm shift or herniation	First CT imaging, done any time during hospital admission

Supplemental Table 2: Prevalence of TBI within the EpiC head injury cohort

TBI Category	Total N	TBI Prevalence	Within TBI Prevalence
No TBI	2518	52.29%	
Mild TBI	842	17.49%	36.66%
Moderate TBI	910	18.90%	39.62%
Severe TBI	545	11.32%	23.73%

Supplemental Table 3: Key Pre-Hospital Events by Survival and TBI Severity

	**No TBI/Mild TBI**			**Moderate TBI**			**Severe TBI**		
	**Survivors**	**Decedents**	**p-value**	**Survivors**	**Decedents**	**p-value**	**Survivors**	**Decedents**	**p-value**
**Primary EMS transport**			0.01*			<0.01^			0.05*
Yes	1425 (43.04%)	30 (61.22%)		385 (43.55%)	21 (80.77%)		168 (61.31%)	188 (69.37%)	
No	1886 (56.96%)	19 (38.78%)		499 (56.45%)	5 (19.23%)		106 (38.69%)	83 (30.63%)	
**Time from injury to first facility arrival**	2 (1.4, 4.1)	1.7 (1.3-2.3)	0.02†	2 (1.2, 3.1)	2 (1.6, 3.5)	0.65†	1.7 (1, 2.2)	1.6 (1, 2.1)	0.68†
**Time to highest level facility**	3.98 (2, 10.8)	2.0 (1.5, 6.9)	<0.01†	9.5 (3.0, 16.0)	5.4 (2.0, 15.2)	0.20†	7.0 (2.0, 12.0)	2.1 (1.3, 7.3)	<0.01†

*chi-squared test

^fisher's exact test

†wilcoxon

Supplemental Table 4: Key Pre-Hospital Events by Survival and TBI Severity

Characteristics	All (N=4815)	Did not encounter TBH (N=2495)	Encountered TBH and did not receive GODS or did not survive (N=1380)	Encountered TBH and received GODS and survived (N=940)
Patient's Sex, n (%)				
Male	4033 (83.8%)	2023 (81.1%)	1172 (84.9%)	838 (89.1%)
Female	782 (16.2%)	472 (18.9%)	208 (15.1%)	102 (10.9%)
Age, median (IQR)	32.0 (26.1, 39.6)	31.9 (26.2, 39.7)	32.3 (26.3, 39.7)	31.7 (25.6, 39.5)
N (Missing)	4815 (0)	2495 (0)	1380 (0)	940 (0)
Substance abuse or Alcohol use disorder, n (%)	499 (10.4%)	352 (14.1%)	75 (5.4%)	72 (7.7%)
GCS Qualifier for worst GCS in first 24 hours, n (%)	33 (0.7%)	8 (0.7%)	16 (2.5%)	9 (2.5%)
Intoxication confirmed or suspected at time of injury n (%)	1524 (31.7%)	846 (33.9%)	375 (27.2%)	303 (32.2%)
Mechanism of Injury, n (%)				
Firearm	222 (4.6%)	53 (2.1%)	118 (8.6%)	51 (5.4%)
Struck/hit	2303 (47.8%)	1248 (50.0%)	548 (39.7%)	507 (53.9%)
Stabbing or cut	1191 (24.7%)	801 (32.1%)	262 (19.0%)	128 (13.6%)
Vehicular Injury	887 (18.4%)	289 (11.6%)	386 (28.0%)	212 (22.6%)
Fall	166 (3.4%)	82 (3.3%)	48 (3.5%)	36 (3.8%)
Other	46 (1.0%)	22 (0.9%)	18 (1.3%)	6 (0.6%)
Injury Force Type, n (%)				
Blunt	3001 (62.3%)	1414 (56.7%)	921 (66.7%)	666 (70.9%)
Penetrating	1220 (25.3%)	776 (31.1%)	315 (22.8%)	129 (13.7%)
Blunt + Penetrating	594 (12.3%)	305 (12.2%)	144 (10.4%)	145 (15.4%)
Intent of Injury, n (%)				
Unintentional (or accidental)	1075 (22.3%)	405 (16.2%)	431 (31.2%)	239 (25.4%)
Intentional: self-harm or suicide	17 (0.4%)	3 (0.1%)	12 (0.9%)	2 (0.2%)
Intentional: assault/homicide	3671 (76.2%)	2069 (82.9%)	916 (66.4%)	686 (73.0%)
Legal intervention/war operations	8 (0.2%)	3 (0.1%)	4 (0.3%)	1 (0.1%)
Undetermined	44 (0.9%)	15 (0.6%)	17 (1.2%)	12 (1.3%)
Primary EMS Transport, n (%)	2217 (46.0%)	1157 (46.4%)	668 (48.4%)	392 (41.7%)
Total Hours* from Injury to First Healthcare Encounter, median (IQR)	2.0 (0.5, 2.9)	2.0 (0.8, 4.0)	1.0 (0.5, 2.0)	1.9 (0.5, 2.3)
N (Missing)	4814 (1)	2494 (1)	1380 (0)	940 (0)
Total Hours* from Injury to First Facility Arrival, median (IQR)	2.0 (1.3, 3.6)	2.1 (1.5, 4.5)	2.0 (1.1, 2.7)	2.0 (1.3, 3.2)
N (Missing)	4814 (1)	2494 (1)	1380 (0)	940 (0)
First Facility Tier, n (%)				
Primary	872 (18.1%)	141 (5.7%)	426 (30.9%)	305 (32.4%)
Secondary District	841 (17.5%)	683 (27.4%)	96 (7.0%)	62 (6.6%)

Secondary Regional	2473 (51.4%)	1671 (67.0%)	478 (34.6%)	324 (34.5%)
Tertiary	629 (13.1%)	0 (0.0%)	380 (27.5%)	249 (26.5%)
Total Hours* from Injury to Highest Facility Arrival, median (IQR)	4.8 (2.0, 12.0)	2.4 (1.7, 5.5)	10.2 (3.6, 16.1)	10.2 (4.3,16.3)
Highest Facility Tier, n (%)				
Primary	12 (0.2%)	12 (0.5%)	0 (0.0%)	0 (0.0%)
Secondary District	621 (12.9%)	621 (24.9%)	0 (0.0%)	0 (0.0%)
Secondary Regional	1862 (38.7%)	1862 (74.6%)	0 (0.0%)	0 (0.0%)
Tertiary	2320 (48.2%)	0 (0.0%)	1380 (100.0%)	940 (100.0%)
Experienced >1 Facility transfer, n (%)	57 (1.2%)	0 (0.0%)	27 (2.0%)	30 (3.2%)
NISS, median (IQR)	9.0 (3.0, 22.0)	3.0 (2.0, 9.0)	12.0 (5.0, 27.0)	22.0 (14.0, 29.0)
N (Missing)	4815 (0)	2495 (0)	1380 (0)	940 (0)
SI >=1.4 within 24 hours of injury, n (%)	115 (3.5%)	38 (3.0%)	52 (4.2%)	25 (3.1%)
Missing	1503	1234	128	141
Highest TEWS Score, median (IQR)	4.0 (3.0, 6.0)	4.0 (3.0, 5.0)	5.0 (3.0, 7.0)	5.0 (3.0, 6.0)
N (Missing)	4815 (0)	2495 (0)	1380 (0)	940 (0)
Worst SATS, n (%)				
Red	1032 (21.4%)	312 (12.5%)	463 (33.6%)	257 (27.3%)
Orange	2470 (51.3%)	1361 (54.5%)	636 (46.1%)	473 (50.3%)
Yellow	1219 (25.3%)	787 (31.5%)	252 (18.3%)	180 (19.1%)
Green	94 (2.0%)	35 (1.4%)	29 (2.1%)	30 (3.2%)
Highest AIS Head Severity Score, median (IQR)	1.0 (1.0, 3.0)	1.0 (1.0, 2.0)	2.0 (1.0, 3.0)	3.0 (3.0, 4.0)
N (Missing)	4665 (150)	2430 (65)	1302 (78)	933 (7)
AIS non-head severity >=3, n (%)	474 (9.8%)	167 (6.7%)	198 (14.3%)	109 (11.6%)
Head CT performed, n (%)	2267 (47.1%)	415 (16.6%)	973 (70.5%)	879 (93.5%)
Head Injury Severity, n (%)				
No TBI	2518 (52.3%)	1819 (72.9%)	660 (47.8%)	39 (4.1%)
Mild TBI	842 (17.5%)	423 (17.0%)	211 (15.3%)	208 (22.1%)
Mod TBI	910 (18.9%)	145 (5.8%)	240 (17.4%)	525 (55.9%)
Sev TBI	545 (11.3%)	108 (4.3%)	269 (19.5%)	168 (17.9%)

TBI = Traumatic Brain Injury; IQR = Interquartile Range; EMS = Emergency Medical Services; NISS = New Injury Severity Score; SI = Shock Index; TEWS = Triage Early Warning Scale; SATS = South African Triage Scale; AIS = Abbreviated Injury Scale; *Total Hours is total hours from injury, inclusive of all transport time and time spent at prior facility(ies), as appropriate

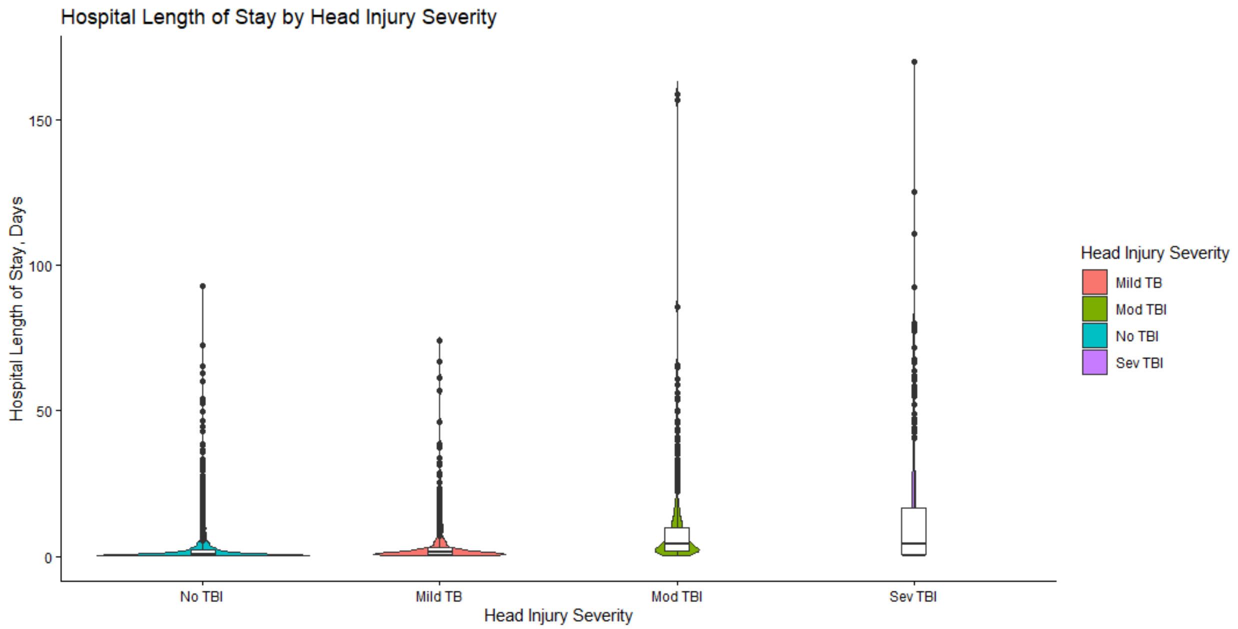

Supplemental Figure 1. Hospital Length of Stay among all patients (days until death, discharge, or lost to follow-up) by TBI Category
